# How Workers Live—The Woollen Industry

**Published:** 1894-07-07

**Authors:** 


### Studies in Applied Sociology.

### XII.—HOW WORKERS LIVE—THE WOOLLEN INDUSTRY.

The woollen industry is, *par excellence*, a British manufacture, and on the whole no foreign country is so fortunate in it. The workers earn comfortable incomes, and it is doubtful if they can improve their circumstances by going abroad. In the Scotch wool industry the wages are particularly good, for although wives do not work there, the family income amounts to £145 per annum. This, however, includes the wages of children, who contribute more than half the sum. In England wages are lower, the earnings of husband, wife, and children making an average of only £103 per family. France and Germany come very much lower, the French family making only £88, and the Germans £57.

In America wages are higher, but not so much so as in other trades; and, indeed, only in two States, New York and Massachusetts, is the Scotch average exceeded. In these the family earnings stand at £154 and £153 respectively. Next to these come Pennsylvania (£140), Maine (£138), and New Hampshire (£131). In Delaware the income falls to £109, and in New Jersey to a trifle less. Taking it all round, therefore, we may infer that the American woollen operative is little better off than the Britisher, though perhaps his income is superior to that of the worker of the Continent. Meanwhile his expenses are higher. For rent in Pennsylvania the family pays £25, as against about £11 in Britain, indulging, however, in an extra room for the money. While this is exceptionally high we find the average rent of a five or six room house in several States—Rhode Island, New York, New Jersey—is £18 or £19. In Maine it is £17, and in Massachusetts this is also the average rent, but less accommodation—not more than four rooms—can be secured for it. Delaware is the cheapest State. There a house of six rooms can be had for £13.

The expenditure for food is generally, but not invariably, larger in America. In New Jersey it falls as low as £36, which is less than the French average (£39), and in Delaware, where it stands at £42, it is less than the British expenditure, which is reckoned at £46. In Maine the expenditure on food is about £1 more than in Britain. The average may be taken at £54, though in Connecticut it rises to £75. The cost of clothing is invariably larger in the States. The average expenditure on this in England is £3 15s. for the husband, £3 10s. for the wife, and £11 for the children, though when we bring this down to a finer analysis we find that the wife of the Scotchman spends about £4 on her own dress and nearly £17 on seeing her "bairns gang braw." The children of the German operative are not so favoured, for though he spends on his own clothing a little more than the Englishman, he allows for his children just £4, and his wife has to make the best appearance she can on £2 10s. The Frenchman spends the same amount as the German on his own clothes, but allows his wife £3 and his children £8.

All through America men's clothes are dear. The lowest sum recorded for this object is in Delaware, where the average is given at £4 10s. The highest is in Massachusetts, where it stands at a little over £6. The average of the other States where wool is manufactured is £5 10s. Working men's clothes bear a distinct proportion to working men's needs; the gratification of vanity in dress is given to—or, as the case may be, withheld from, wives and children. Thus we found the expenditure of the men in France, Germany, and England identical, though very varying sums were spent on their families. We may infer, therefore, that there is no great prosperity where these do not get a fair share of the family income for dress. We find that the American woman generally does get her fair share, but in most cases it is less in proportion than that of her British, and especially her Scottish, sister. She never, like the latter, has the delight of spending more than her good man; but in Maine and New Jersey she has as much—£6 in the first, and £5 in the latter. In Connecticut it is maternal vanity that is strongest, and while the wife spends no more than £3 10s. on herself, she gives over £17 to her children. Next in devotion come the mothers of New York, who spend £4 on themselves and £12 on their offspring. In Pennsylvania the husband has £5 10s., the wife £4 10s., and the children £11. The New Jersey matron gives £8 to her children, as against £5 to herself; but in Rhode Island the children get only 8s. less, while the mother reduces her own expenditure to £3 12s. Taking the American average all over, we may say that the man spends £5 10s. in clothes, the wife £4 5s., and the children £11 10s.

In the expenditure on religion and charity national characteristics show themselves. Thus the Englishman gives £1 3s. to his church, and £1 2s. to alms. The Scotchman gives £1 15s. to religion, but reduces his charity to 10s. 6d. Sceptical France gives, indeed, 13s. to the church, but to charity 17s.; while the prudent German endows his church to the extent of 5s., and bestows in charity nothing at all. The American gives generously to religion; in New Hampshire, New York, and Connecticut £2 5s. to £2 10s.; and over all the wool manufacturing States an average of £1 15s.; but charity comes off but moderately with an average of 13s. 6d. On furniture and books the American spends about twice as much as the European, but in amusements only half. In this item the Frenchman leads with £5 per family, the Englishman follows hard with £4 10s.; while even in New York, which seems the most luxurious of the States, the expenditure does not exceed £3, and in sober Massachusetts it is as low as 13s. 6d. The workman's drink bill is heaviest in New York—£7 10s., but that is due more to the Canadian and the Scotchman than to the native-born American; next comes France with £7, then Britain with £3 6s. The lowest sum per family is in Massachusetts, where it amounts to only 10s. 6d.; but one cannot help wondering how, in a prohibition State like Maine, the workman's account for intoxicants comes to be set down at £2 7s. 6d.

